# A pre‐investigational new drug study of lung spheroid cell therapy for treating pulmonary fibrosis

**DOI:** 10.1002/sctm.19-0167

**Published:** 2020-04-18

**Authors:** Jhon Cores, Phuong‐Uyen C. Dinh, Taylor Hensley, Kenneth B. Adler, Leonard J. Lobo, Ke Cheng

**Affiliations:** ^1^ Joint Department of Biomedical Engineering University of North Carolina, and North Carolina State University Chapel Hill and Raleigh North Carolina USA; ^2^ Department of Molecular Biomedical Sciences and Comparative Medicine Institute North Carolina State University Raleigh North Carolina USA; ^3^ Division of Pulmonary Diseases and Critical Care Medicine University of North Carolina Chapel Hill North Carolina USA; ^4^ Division of Pharmacoengineering and Molecular Pharmaceutics University of North Carolina Chapel Hill North Carolina USA

**Keywords:** cell therapy, idiopathic pulmonary fibrosis, lung spheroid cells, preclinical

## Abstract

Idiopathic pulmonary fibrosis is a lethal interstitial lung disease with unknown etiology, no cure, and few treatment options. Herein, a therapy option is presented that makes use of a heterogeneous population of lung cells, including progenitor cells and supporting cells lines, cultured in adherent and suspension conditions, the latter of which induces spontaneous spheroid formation. Within these spheroids, progenitor marker expression is augmented. The cells, called lung spheroid cells, are isolated from fibrotic lungs, expanded, and delivered in single cell suspensions into rat models of pulmonary fibrosis via tail‐vein injections. Two bleomycin‐induced fibrotic rat models are used; a syngeneic Wistar‐Kyoto rat model, treated with syngeneic cells, and a xenogeneic nude rat model, treated with human cells. The first objective was to study the differences in fibrotic progression in the two rat models after bleomycin injury. Nude rat fibrosis formed quickly and extended for 30 days with no self‐resolution. Wistar‐Kyoto rat fibrosis was more gradual and began to decrease in severity between days 14 and 30. The second goal was to find the minimum effective dose of cells that demonstrated safe and effective therapeutic value. The resultant minimum effective therapeutic dose, acquired from the nude rat model, was 3 × 10^6^ human cells. Histological analysis revealed no evidence of tumorigenicity, increased local immunological activity in the lungs, or an increase in liver enzyme production. These data demonstrate the safety and efficacy of lung spheroid cells in their application as therapeutic agents for pulmonary fibrosis, as well as their potential for clinical translation.

## Graphical abstract

Lung spheroid cells are a cellular therapeutic achieved after a sequential 2D‐3D‐2D culture regiment. The steps include acquiring transbronchial biopsies from human idiopathic pulmonary fibrosis (IPF) patients, allowing the cells to migrate out of the biopsy, creating spheres from the migrated cells in suspension culture, and replating the spheres, which adhere and separate into individual LSCs.

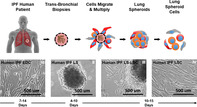

**Significance statement**

This study helps set the stage for the transition of this cellular therapy paradigm from rodent models to clinical trials in a number of ways. The transbronchial acquisition of the lung spheroid cells is a minimally invasive strategy suitable for the clinic and preferable to thoracoscopic alternatives; the doses used in this study are scalable, manufacturable, and comparable to currently existing clinical trial efforts targeting other lung diseases; and the intravenous route of administration used is applicable to clinical trials, as it presents an easy, quick, and patient‐friendly way to administer the treatment.

## 1 INTRODUCTION

The lungs are constantly exposed to ambient toxins, noxious gases, and infectious pathogens. When their natural repair mechanisms^1^ are damaged by repeated injury, pathologies such as idiopathic pulmonary fibrosis (IPF) can develop.^2^ IPF is a devastating, restrictive interstitial lung disease characterized by usual interstitial pneumonia and lung architecture remodeling, leading to heterogeneous extracellular matrix (ECM) deposition.^3^, ^4^ Although fibrosis is not a phenomenon exclusive to the lungs, their exposure to environmental, chemical, and biological insults make them especially susceptible.^5^ IPF is chronic, usually progresses at a gradual pace, and is ultimately fatal. Most patients present for diagnosis at an advanced stage and receive a bleak prognosis, with median patient life expectancy falling to within 2 to 3 years.^6^

Animal models of IPF have advanced the scientific understanding of the pathology and allowed researchers to explore treatment options not currently available. Among the many fibrotic agents/drugs that have been used to create animal models, including radiation,^7^ silica,^8^ asbestos,^9^ and transgenic manipulation,^10^ bleomycin is the most consequential and most commonly used. It is a chemotherapeutic antibiotic that is believed to disrupt the cellular cycle by cleaving single‐ and double‐stranded DNA.^11^ The rodent models created with it demonstrate high reproducibility, accessibility, and histological similarity to the real disease.^12^ However, each animal species and breed reacts differently to the bleomycin injury.^13^ Therefore, fibrosis may take longer to manifest in some rodent models than in others.

Herein, two rat breeds were used to explore differences in the onset of bleomycin‐induced pulmonary fibrosis (PF). After disease onset, the rodents were injected with a cellular therapy with the hope of establishing therapeutic safety and efficacy. The cells used were derived from either rodent lung tissue samples or human lung biopsies, expanded as heterogeneous cell populations in vitro, and injected into the PF rodent models. By obviating cell sorting, the progenitor cells present in the extracted population were cultured with supporting cell lines that offer a more biologically relevant in vitro environment. To obtain cells, tissue samples were processed using a three‐stage adherence‐suspension‐adherence culture method. During the first adherence stage, cells migrate out from the tissue samples; these are termed explant‐derived cells (EDCs). The EDCs are then seeded onto ultra‐low attachment flasks; under these culture conditions, the outgrowth cells spontaneously form three‐dimensional (3D) cell agglomerations in suspension, termed lung spheroids (LSs). During this stage, the cells exist in a biomimetic 3D environment that has been shown to enhance their expression of progenitor markers.^14^ Other labs have used variations of the spheroid/organoid culture technique to study cellular interactions, lung development, and lineage tracing.^15^, ^16^, ^17^, ^18^, ^19^, ^20^, ^21^ When plated onto fibronectin‐coated surfaces, these LSs generate cells that we term lung spheroid cells (LSCs). Cells were injected into the rodent models at the LSC stage.

Previously, we demonstrated the safety and efficacy of human lung spheroid cells (hLSCs) explanted from healthy lungs in a severe combined immunodeficient mouse model of PF.^22^ We have shown the potential for an allogenic variant of this therapy using Wistar‐Kyoto (WKY) and Brown Norway rats.^23^ In addition, we have verified the feasibility of acquiring LSCs from minimally invasive transbronchial biopsies.^14^ Herein, we have developed a clinically viable therapeutic strategy using native LSCs, explanted from fibrotic lungs, to treat PF in rodent models. To this end, two studies were conducted using an immunocompetent inbred WKY rat model and an immunocompromised outbred nude rat mode. After fibrotic onset, the WKY rats were injected with LSCs derived from bleomycin‐induced, fibrotic WKY rat lungs (rLSCs). The WKY rat model was used to test the safety and efficacy of an autologous cell transplantation in diseased rats. The nude rats were injected with cells derived from the transbronchial lung biopsies of human IPF patients. The nude rat model was used to test the therapeutic safety and efficacy of the human IPF‐derived LSC cell line.

In the first study (Bleomycin‐induced pulmonary fibrosis progression study), the progression of bleomycin‐induced PF in both rat breeds was tracked. Although pathology timelines have been reported for rodents in general, it is important to understand the breed‐specific transition point between inflammation and fibrosis and the extent/duration of fibrosis. The fibrotic timelines would then dictate the timing of LSC injections. Bleomycin‐induced fibrosis in rodents begins with an acute inflammatory phase which gives way to stable fibrotic manifestation. Knowing the point of transition between inflammation and fibrosis ensured that the cells were infused after fibrosis had matured to better reflect the clinical condition of patients who are diagnosed with IPF. Periods of peak inflammation, peak fibrosis, and eventual self‐resolution were explored, ensuring a therapeutic regimen that ended before partial reversibility of bleomycin‐induced fibrosis could occur.^24^, ^25^

In the second study (dose finding and safety study), dosing experiments were conducted to (a) establish the minimum effective dose (MED) required for efficacy and (b) evaluate the safety of the MED based on resultant organ tumorigenicity, liver enzyme production, in situ immune reactions, and rodent adverse events. This second study was organized into three dosing groups (1 × 10^6^, 3 × 10^6^, and 5 × 10^6^ LSCs) that were analyzed 10 days after bleomycin infusion.

Taken as a whole, this work has contributed to the development of a soon‐to‐start phase I, standard‐care‐controlled, dose escalation clinical trial, which will examine the safety and efficacy of the intravenous injection of autologous LSCs in patients with IPF.

## 2 RESULTS

### 2.1 Inflammatory responses in nude and WKY rats

**FIGURE 1:**
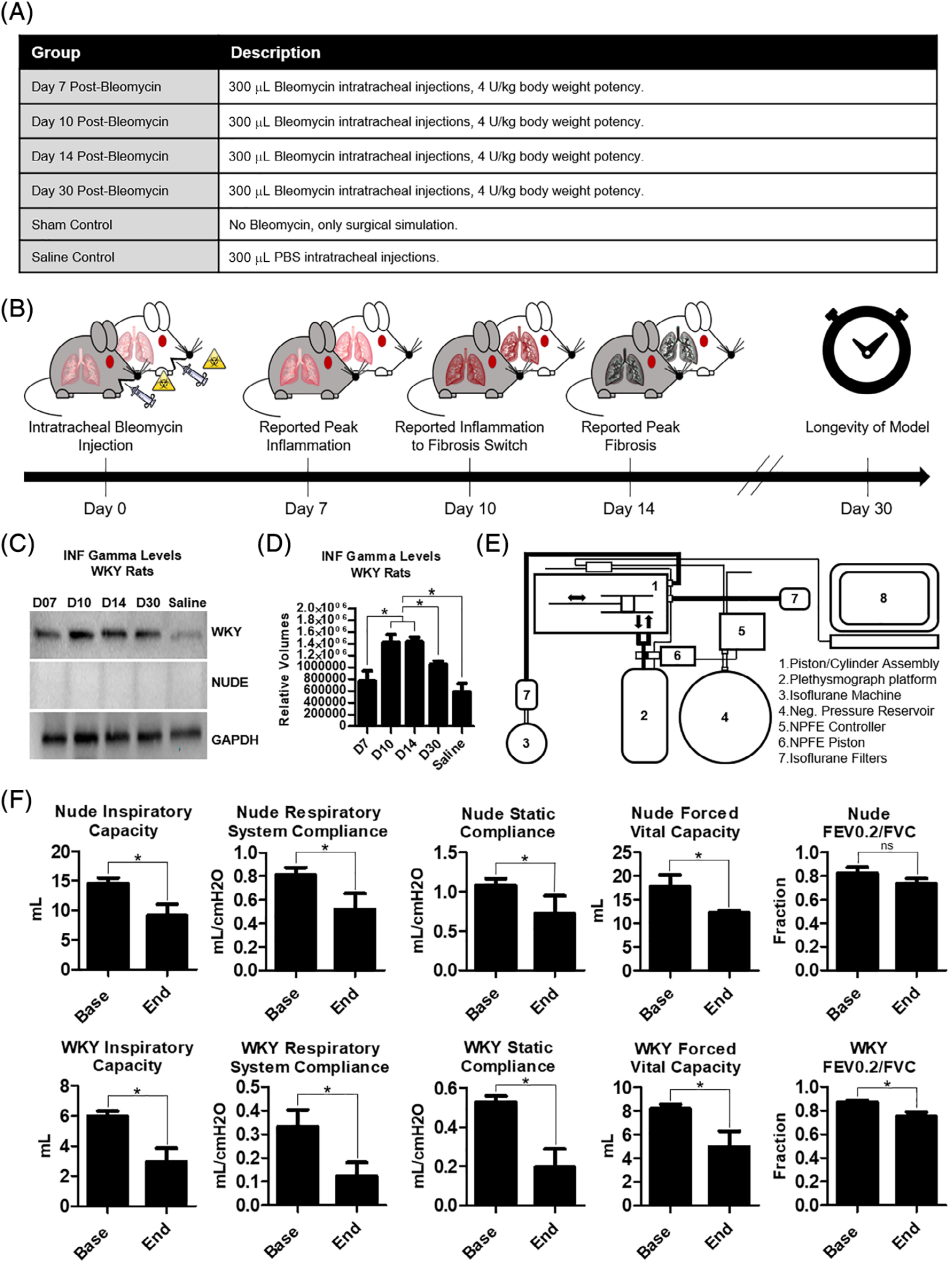
Inflammatory and functional properties of the bleomycin rodent models of pulmonary fibrosis. A, A summary of the six groups analyzed and compared in the fibrosis progression study. Female nude and WKY rats were used. B, Literature reported estimated timeline of bleomycin‐generated pulmonary fibrosis in rodents. This timeline is the standard which was used to compare the nude (gray) and the WKY rats (white). C, Western blot results: INF gamma levels in WKY and nude rat lung protein isolates. D, Bar graph summary of Western blot results for WKY rats, n = 4. E, Schematic of the small‐animal ventilator and plethysmograph used to assess live lung function in the rodent models. F, Bar graph panels summarizing the functional lung parameters in the rodent models, n = 3‐4. Base values were taken before bleomycin injections. End values were taken 30 days after bleomycin injections. Statistical analysis: D (one‐way analysis of variance with Bonferroni's multiple comparison test); F (Student's *t* test with a 95% confidence interval). Error bars represent SD. *P ≤ .05. FEV0.2, forced expiratory volume at 0.2 seconds; FVC, forced vital capacity; GAPDH, glyceraldehyde 3‐phosphate dehydrogenase; INF, interferon; NPFE, negative pressure forced expiration; ns, no significant difference; WKY, Wistar‐Kyoto

**FIGURE 2:**
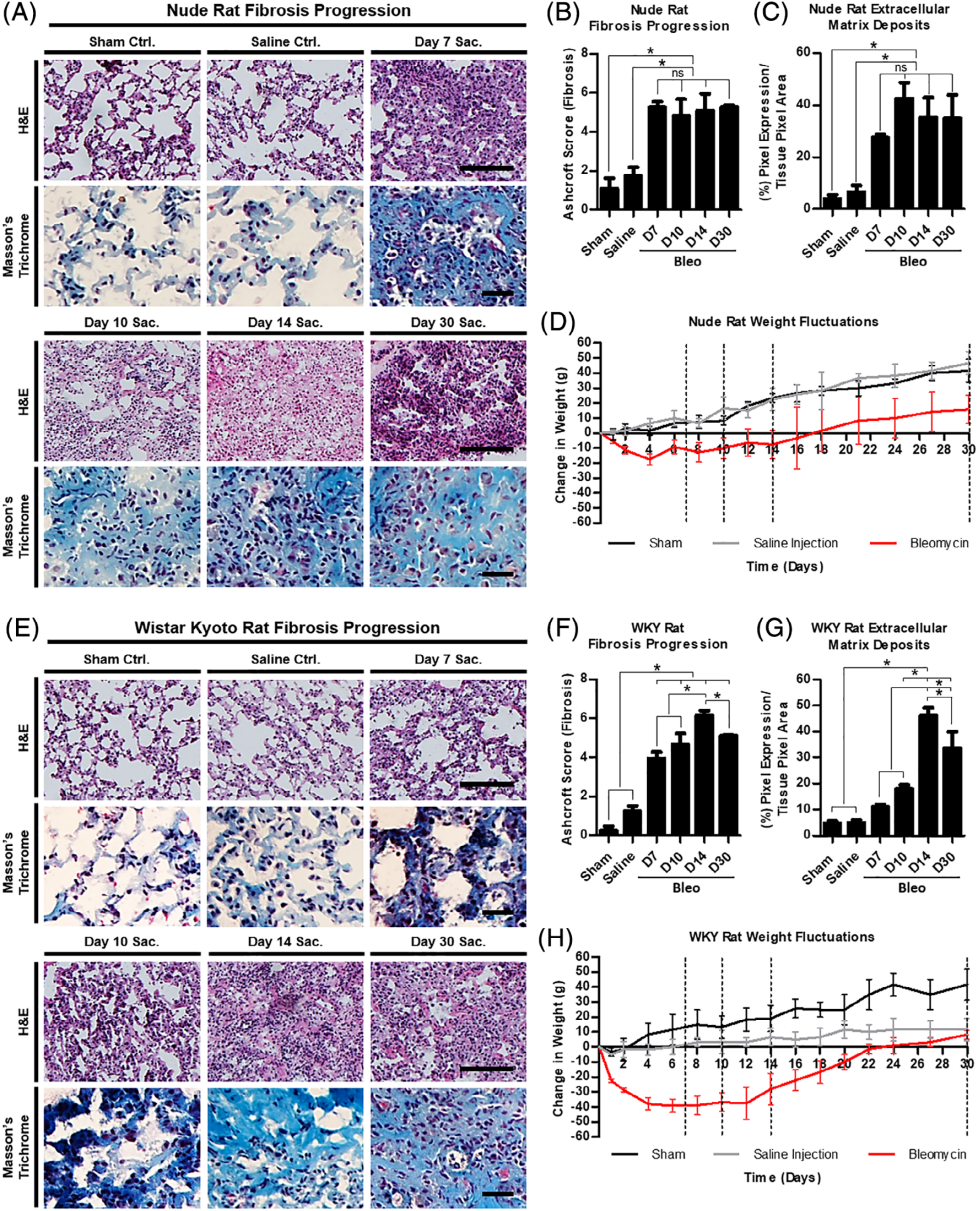
Manifestation of pulmonary fibrosis. A, Bright field micrographs of representative lung sections from each nude rat study group stained with H&E and Masson's trichrome. B, Bar graph comparing the Ashcroft scores between each group in the nude rat model. C, Bar graph comparing the extracellular matrix deposits contributing to the nude rat pulmonary fibrosis. D, Line graph summarizing the weight fluctuations observed in the nude rats intratracheally injected with bleomycin, saline, or nothing at all. E, Bright field micrographs of representative lung sections from each WKY rat study group stained with H&E and Masson's trichrome. F, Bar graph comparing the Ashcroft scores between each group in the WKY rat model. G, Bar graph comparing the extracellular matrix deposits contributing to the WKY rat pulmonary fibrosis. H, Line graph summarizing the weight fluctuations observed in the WKY rats intratracheally injected with bleomycin, PBS, or nothing at all. Scale bars (H&E) = 100 μm, (trichrome) = 25 μm. Statistical analysis: one‐way analysis of variance with Bonferroni's multiple comparison test, n = 3. Error bars represent SD. *P ≤ .05. H&E, hematoxylin and eosin; ns, no significant difference; Sac., sacrifice; WKY, Wistar‐Kyoto

The inflammatory response was analyzed to assist in tracking the fibrotic progression and the point of transition between inflammation and fibrosis after bleomycin injury. Group assignments are organized in Figure 1A. Using the saline group as a control, protein was extracted from the fibrotic rat lungs and tested for interferon gamma levels (IFN‐γ) (Figure 1C,D) via Western blot electrophoresis. IFN‐γ upregulates the adhesion molecules and chemokines that induce the migration of key immune cells to sites of inflammation.^26^ The largest concentrations of the inflammatory protein were found on days 10 (1.43 × 10^6^ ± 1.31 × 10^5^) and 14 (1.44 × 10^6^ ± 7.52 × 10^4^) after bleomycin injection (Figure 1D). A full summary of all inflammatory values is provided in Supplemental Figure S4. Early published research suggested a switch between inflammation and fibrosis around day 9 after bleomycin injection (Figure 1B).^27^ However, this research was done on WKY, not nude rats. Moreover, our data show a congruence between the peak fibrosis (Figure 2E‐G) and peak inflammation time points (Figure 1C,D), indicative of a dynamic process, in which both fibrosis and inflammation overlap as the pathology evolves. Current research suggests PF is not beholden exclusively to the ebb and flow of inflammation, but rather, to defects in the organ's ability to self‐heal due to fibroblast/epithelial cell dysfunction.^28^ In addition, although there may be an inflammatory response present in nude rats, there was no detectable IFN‐γ presence in the athymic nude rat lungs. Furthermore, the fibrotic timeline observed in the nude rats (Figure 2A‐C) provides enough information to ensure that therapeutic cells would be administered during the fibrotic phase of the disease.

### 2.2 Functional changes as a result of bleomycin injury

Important functional respiratory parameters were analyzed using a small‐animal ventilator and plethysmography chamber (Figure 1E). These were inspiratory capacity (IC), respiratory system compliance (Crs), static compliance (Cs), forced vital capacity (FVC), and the fraction of forced expiratory volume at 0.2 seconds (FEV_0.2_), in relation to the FVC. The data output further highlighted the differences between the WKY and nude rodent models.

IC is the amount of air that can be inhaled after a normal exhale. Compliance is a measure of the lung's ability to expand as a result of air pressure. Respiratory system compliance measures the sum of the compliance of the lungs and the chest wall. Static compliance measures the lung's compliance during a period of no air flow, such as when you transition from inhale to exhale, and vice versa. FVC is the total amount of air that can be forcibly exhaled after inhaling the largest quantity of air possible. FEV_0.2_ is the amount of air that can be forced out by a patient in 0.2 seconds. The FEV_0.2_ over FVC ratio measures the obstructive vs restrictive nature of the lung disease produced.^29^ In obstructive lung disease, the total lung capacity remains essentially the same, whereas the rate of outflow is hindered by whatever is obstructing the airways. Thus, FEV_0.2_ should decrease, and with it, the FEV_0.2_/FVC parameter. In restrictive lung diseases, especially those of fibrotic nature, the total lung capacity decreases due to decreased lung compliance, but so does the FEV_0.2_ because it is harder for a patient to force air out of less compliant lungs. Since IPF is primarily a restrictive lung disease, all of the parameters analyzed are expected to decrease in a diseased state, with the exception of the FEV_0.2_/FVC value, which may remain the same or even increase. This occurs when the decrease in lung capacity outpaces the decrease in a patient's ability to force air out of their lungs.^30^

We compared the baseline states with the endpoint states of each rat in the day 30 excision group, as well as between the rodent breeds at both time points (Figure 1F). All the following respiratory parameters experienced a decrease in value as the fibrosis progressed from baseline to endpoint in the WKY rats: IC (5.985 ± 0.338 vs 2.932 ± 0.929), Crs (0.334 ± 0.070 vs 0.123 ± 0.058), Cs (0.528 ± 0.030 vs 0.196 ± 0.0922), FVC (8.206 ± 0.356 vs 4.970 ± 1.341), and forced expiratory volume to FVC ratio (FEV0.2/FVC) (0.872 ± 0.015 vs 0.750 ± 0.037). In the nude rats all functional parameters decrease from baseline to endpoint, except the FEV0.2/FVC ratio: IC (14.580 ± 0.956 vs 9.118 ± 1.934), Crs (0.811 ± 0.063 vs 0.519 ± 0.135), Cst (1.081 ± 0.084 vs 0.721 ± 0.228), FVC (17.710 ± 2.460 vs 12.210 ± 0.489), and FEV0.2/FVC (0.822 ± 0.053 vs 0.734 ± 0.046). The difference in FEV0.2/FVC trend between WKY and nude rats highlights the inherent difference that can exist in disease models as a result of breed variation. Both breeds experience a decrease in FVC. However in the nude rats, the decrease in FVC is matched by the decrease in FEV_0.2_, which is why, despite a nominally lower FVC value, the FEV_0.2_/FVC fraction does not significantly change from the baseline. The same is not true in the WKY rats, where the decrease in FVC is outpaced by the decrease in FEV_0.2_.

### 2.3 Fibrotic manifestation in nude and WKY rats

Using the saline and sham control groups as baselines, the progression of bleomycin‐induced fibrosis was tracked using the Ashcroft scoring system,^31^ which defines characteristics of the fibrotic lung pertaining to alveolar septa and lung structure. Fibrotic progression was also tracked through the analysis of collagen/ECM deposition. The nude rat model deviates from the predicted pathological pattern as cited in the literature,^27^ rising rapidly and peaking at day 7 with an Ashcroft score of 5.272 ± 0.278 (Figure 2A,B). From day 7 to day 30, the fibrotic scores hold steady, reflecting a relatively stable fibrosis that persists past the 30‐day endpoint, 5.281 ± 0.096 (Figure 2B). Likewise, ECM deposits become apparent on day 7 and last through day 30 (Figure 2A,C). The WKY rat model more closely follows the predicted pathological pattern, rising steadily until it peaks at an Ashcroft score of 6.154 ± 0.253 at day 14 after bleomycin infusion (Figure 2E,F). Despite some self‐resolution, there is still a significant fibrotic manifestation present at day 30, 5.117 ± 0.057 (Figure 2F). Unlike the nude rat model, the WKY rat lungs begin to suffer significant ECM deposition at day 10 (Figure 2E,G). Thus, the WKY rat fibrotic onset is more gradual than that of the nude rats.

### 2.4 Weight fluctuations

The weight of the rats was tracked and analyzed during the predicted transition periods (days 7, 10, and 14) and through the end point (day 30) in an attempt to discern correlations between weight fluctuations and the pathological evolution of the fibrosis. As shown in Figure 2D, the nude rats lost most of their weight within the first 3 to 4 days after bleomycin infusion, then remained generally stable until day 16 to 18, after which they experienced steady weigh gains. Their prebleomycin weights were reached on day 17. The WKY rats lost most of their weight within the first week after bleomycin infusion, then remained generally stable until day 14, after which they began to regain weight. Their prebleomycin weights were reached 23 days after bleomycin (Figure 2H).

### 2.5 Growth rates and antigenic profile of human and rat LSCs

**FIGURE 3:**
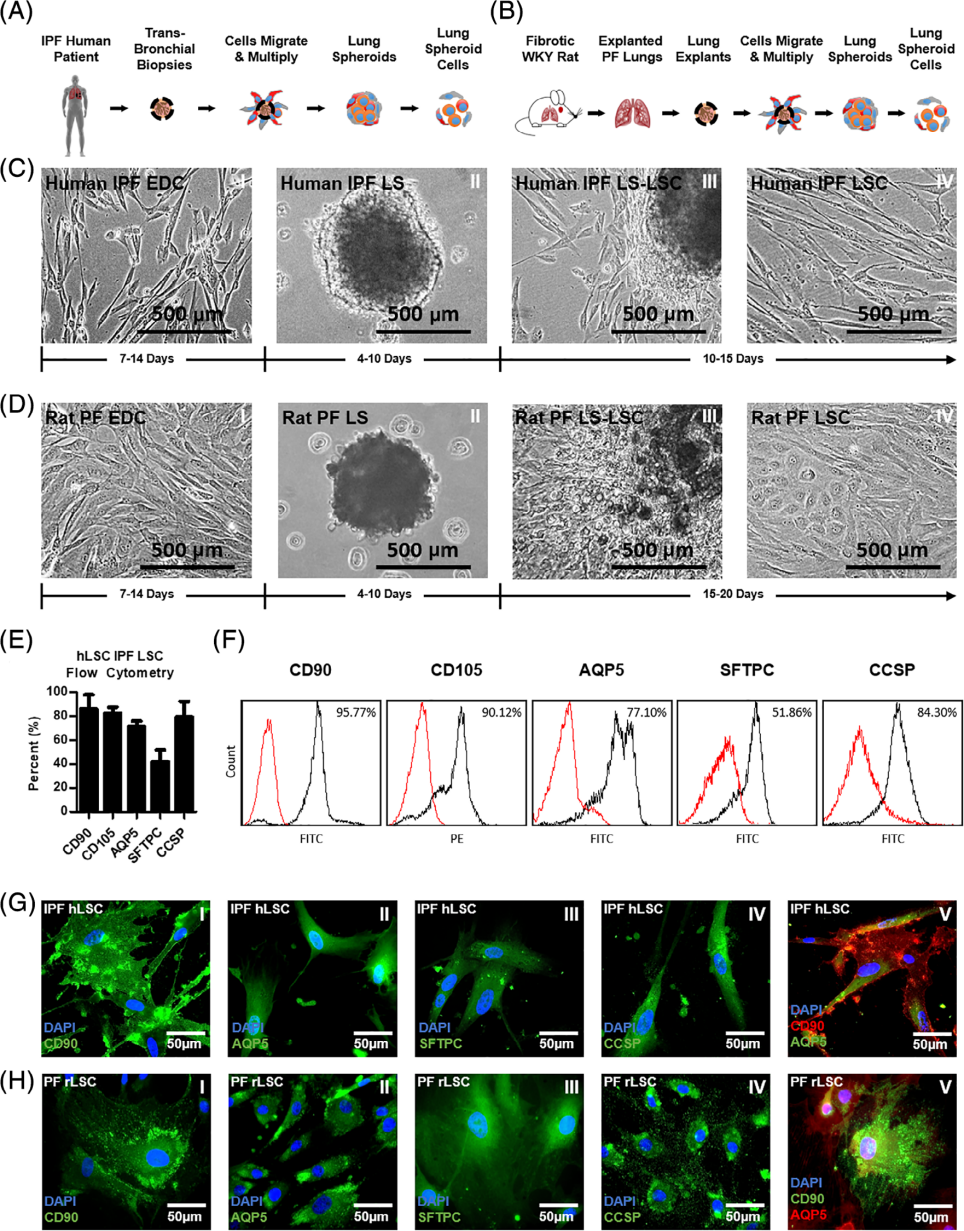
Lung spheroid cell generation and phenotyping. A,B, Illustrations of the stages involved in human and WKY rat LSC culture, respectively. C,D, Bright field micrographs of the different stages involved in human and rat LSC expansion, respectively, and their average developmental timeframes. E, Bar graph summarizing the expression of the human LSC markers analyzed through flow cytometry. F, Representative histogram panel of flow cytometry data collected from human LSCs. G,H, Representative fluorescent micrographs showing the presence of human and rat LSC markers. Error bars represent SD. AQP5, aquaporin 5; CCSP, club cell secretory protein; CD, cluster of differentiation; EDC, explant‐derived cells; hLSC, human LSC; IPF, idiopathic pulmonary fibrosis; LS, lung spheroids; LS‐LSC, transition from LS to LSC stage; LSC, lung spheroid cells; rLSC, rat LSC; PF, pulmonary fibrosis; SFTPC, surfactant protein C

The stages of cell growth and expansion are illustrated in Figure 3A,B. Bright field micrographs of these stages are provided in Figure 3C for human cells, and Figure 3D for rat cells, along with their respective cell culture expansion timelines. For the cell quantities required to conduct our studies, the human cells were ready for injections after 30 days, and the rat cells were ready after 35 days. Human IPF LSCs, expanded from an IPF fibrotic lung, express cluster of differentiation 90 (CD90) (86.1%), cluster of differentiation CD105 (82.2%), surfactant protein C (SFTPC) (41.7%), club cell secretory protein (CCSP) (79.2%), and aquaporin 5 (AQP 5) (71.2%), as verified by flow cytometry (Figure 3E). A subset of the flow cytometry data is presented in Figure 3F, and verified by immunocytochemistry (Figure 3G‐I‐V). Very little cluster of differentiation 45 (CD45) (4.7%) is expressed (Figure S5). The markers were chosen, first, to identify progenitor cell populations in the lung cell cocktail (SFTPC and CCSP) and second, to identify supporting cell lines (CD105, CD90, and AQP5) that can make the environment within which the progenitor cells are cultured more like the environment in the lungs. An in‐depth flow cytometry characterization of hLSCs is available in Dinh et al.^14^ The hLSC cell line had a doubling time of 2.5 days during the LSC stage. The phenotypic markers carry through to the rat IPF LSCs (Figure 3H‐I‐V). The rat LSC cell line had a doubling time of 5.7 days.

### 2.6 Manufacturing feasibility of LSCs from IPF lungs

A summary clinical cell‐expansion protocol is provided in Figure S6. It illustrates the cell growth stages and safety checkpoints needed to undergo an autologous cell infusion procedure. The cells undergo a sequential 2D‐3D‐2D culture system that provides a more biomimetic growth environment which enhances progenitor marker (CCSP and SFTPC) expression (Figure S7). Once the cells transition from the LS to the LSC stage, and before LSC passage 1 (P1), they will be subject to an in‐process safety inspection, which will be repeated at the end of LSC expansion, once the clinically appropriate cell doses of 100 × 10^6^ and 200 × 10^6^ cells are reached. The safety inspection will consist of a series of sterility, mycoplasma, endotoxin, purity, and viability tests. The minimum viability required is 70% of the total cell population, based on the results of a 30‐hour study of LSC viability in Heparin + PBS solution. A summary of human LSC expansion to over 200 million cells, and their viability, is provided in Figure S8.

### 2.7 Removal of fetal bovine serum, collagenase, and gentamicin from the final LSC product

An important prerequisite for cell therapy studies intended for clinical patients is the removal of undefined animal products such as fetal bovine serum (FBS), harsh enzymes such as collagenase, and antibiotics such as gentamicin. Gentamicin will be used for the EDC and LS stages of cell growth, but will not be used as a cell culture antibiotic once the cells reach the LSC stage. Before the final LSC product can be administered to patients, the cells must be washed a minimum of three times with PBS, which removes the FBS content (Figures 9 and S10). The same washing procedure eliminates collagenase from the media, initially used to expand the cells from the biopsy during the EDC stage (Figure S11).

### 2.8 LSC dose determination in nude and WKY rats

**FIGURE 4:**
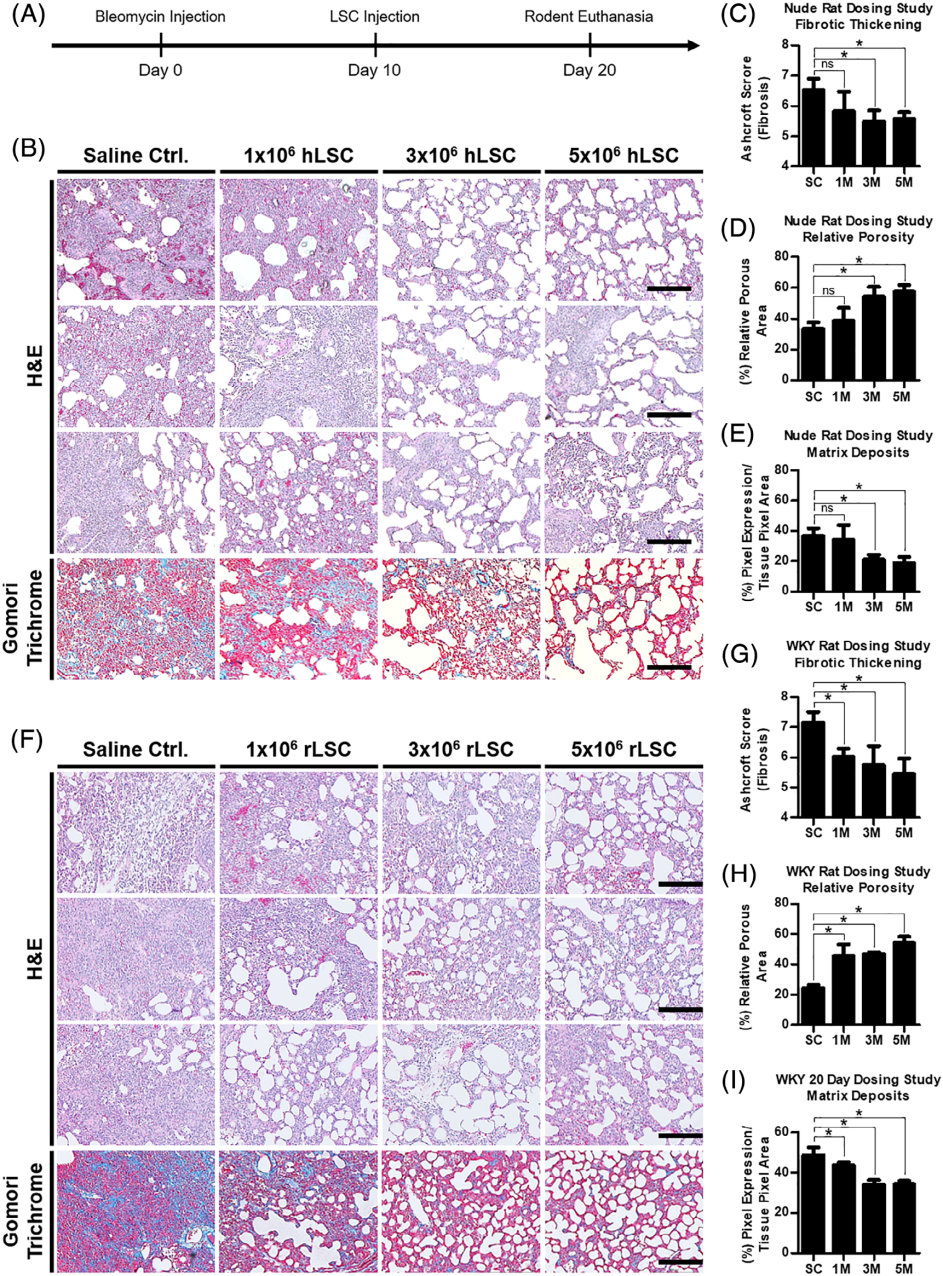
Efficacy of dose scale‐up study in rodents. A, Timeline of dosing study procedures. B, High power, bright field micrograph panel of nude rat lung tissues demonstrating their characteristic, dose‐dependent fibrotic thickening (H&E), porosity (H&E), and extracellular matrix deposits (trichrome), n = 3. C, Bar graph summarizing the Ashcroft scores from the nude rat H&E panel. D, Bar graph summarizing the relative porosity of the lung sections from the nude rat H&E panel. E, Bar graph summarizing the expression of blue pixels (collagen) in nude rat lung tissues stained with Gomori trichrome (bottom row of the histology panel). F, High power, bright field micrograph panel of WKY rat lung tissues demonstrating their characteristic, dose‐dependent fibrotic thickening (H&E), porosity (H&E), and extracellular matrix deposits (trichrome), n = 3. G, Bar graph summarizing the Ashcroft scores from the WKY rat H&E panel. H, Bar graph summarizing the relative porosity of the lung sections from the WKY rat H&E panel. I, Bar graph summarizing the expression of blue pixels (collagen) in WKY rat lung tissues stained with Gomori trichrome (bottom row of the histology panel). Scale bars = 200 μm. Statistical analysis: Student's *t* test with a 95% confidence interval. Error bars represent SD. *P ≤ .05. M, million; Ctrl., control; ns, no significant difference; WKY, Wistar‐Kyoto

A timeline of the procedures involved in the dosing study is provided in Figure 4A. For the nude rats, both the 3 and 5 million doses were effective at reducing fibrotic thickening scores. Three million is the MED and is the dose we will translate to clinical trials after scaling up to appropriate human doses (Figure S12). The 1, 3, and 5 million hLSC doses were compared with the saline control (PBS tail injection) (Figure 4B‐E). The average Ashcroft score for the saline control group was 6.53 ± 0.22, which was considerably higher than the score obtained by the 3 (5.49 ± 0.21) and 5 million cells group (5.57 ± 0.13) (Figure 4C). The 1 million cells group did not achieve statistically significant improvement in Ashcroft score (5.82 ± 0.38), despite trending toward a decrease in fibrotic thickening (Figure 4B,C). These data are further supported by the quantification of the relative porosity of the lung sections (Figure 4D) and the deposition of ECM (Figure 4E). For the WKY rats, all three doses were effective at reducing fibrotic thickening scores. The 1, 3, and 5 million rat LSC doses were compared to the saline control (Figure 4F‐I). The average Ashcroft score for the saline control group was 7.15 ± 0.36, which was considerably higher than the score obtained by the 1 (6.03 ± 0.27), 3 (5.57 ± 0.62), and 5 million cells group (5.45 ± 0.51) (Figure 4G). For the WKY rats, all the doses were effective but no MED was attained. The data are further supported by the quantification of the relative porosity of the lung sections (Figure 4H) and the deposition of ECM (Figure 4I). The MED for the proposed human trial was converted from the nude rats because they received human cells from IPF patients, which are the same cells that will be used in human trials.

### 2.9 Tumorigenicity studies of LSC therapy

**FIGURE 5:**
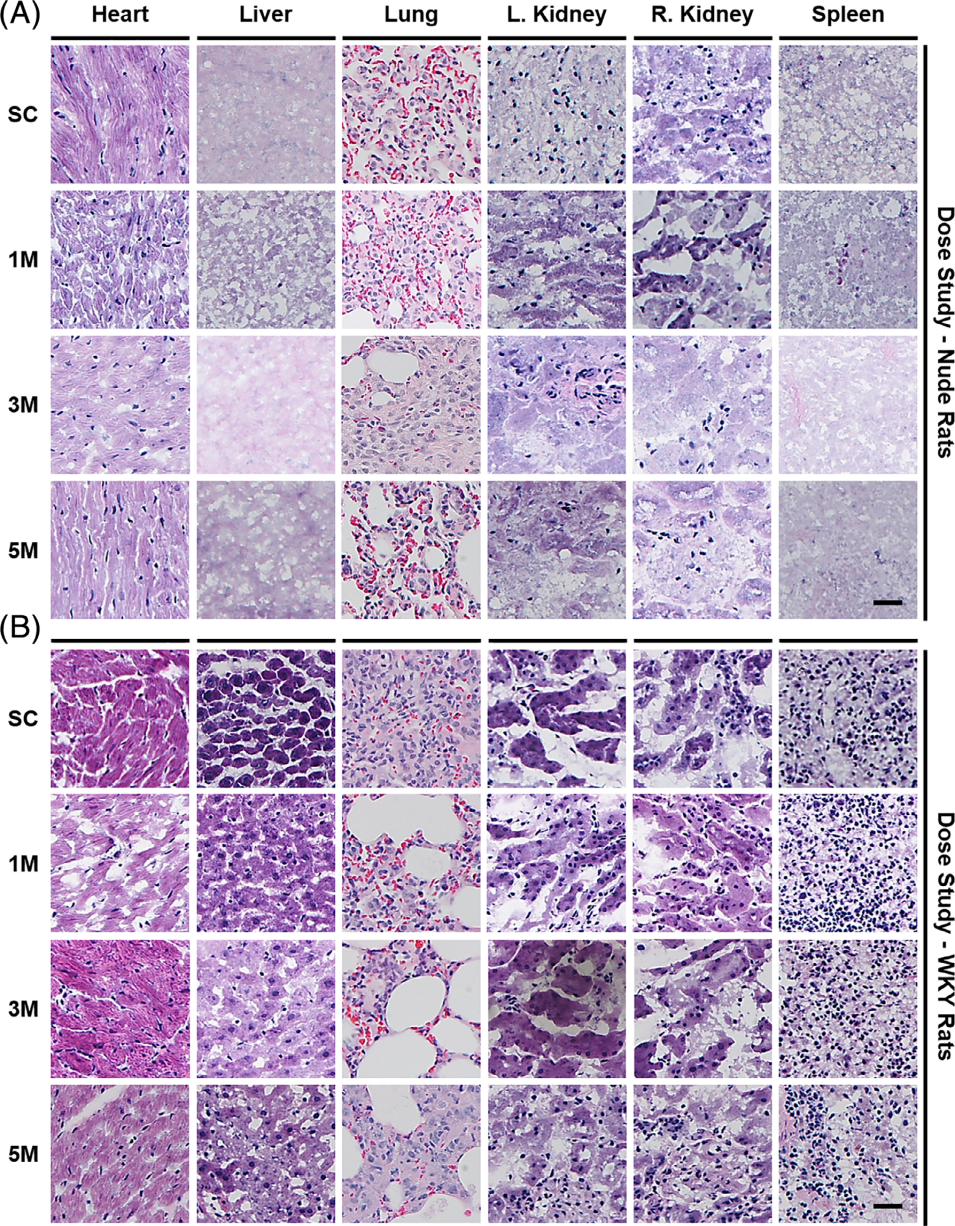
Safety study; tumorgenicity assessment of LSC injections. A, Representative bright field micrographs of H&E stained organ samples from nude rats. B, Representative bright field micrographs of H&E stained organ samples from WKY rats. Scale bars = 25 μm. SC, saline control; M, million; WKY, Wistar‐Kyoto

We took a careful look at any adverse events that might have occurred at different time points throughout the study (Figures S13 and S14). We then conducted a histological examination of the organs of the nude and WKY rats after euthanasia to look for the presence of tumors (Figure 5A,B, Figures S15 and S16). No evidence of tumor formation was found in any of the rats studied. This, in combination with our previous studies in rats, and our overdosing safety studies in nude mice, provides a track record of safety as we scale up the therapy and transition into human clinical trials.^14^, ^22^, ^23^

### 2.10 Toxicity studies of LSC therapy

**FIGURE 6:**
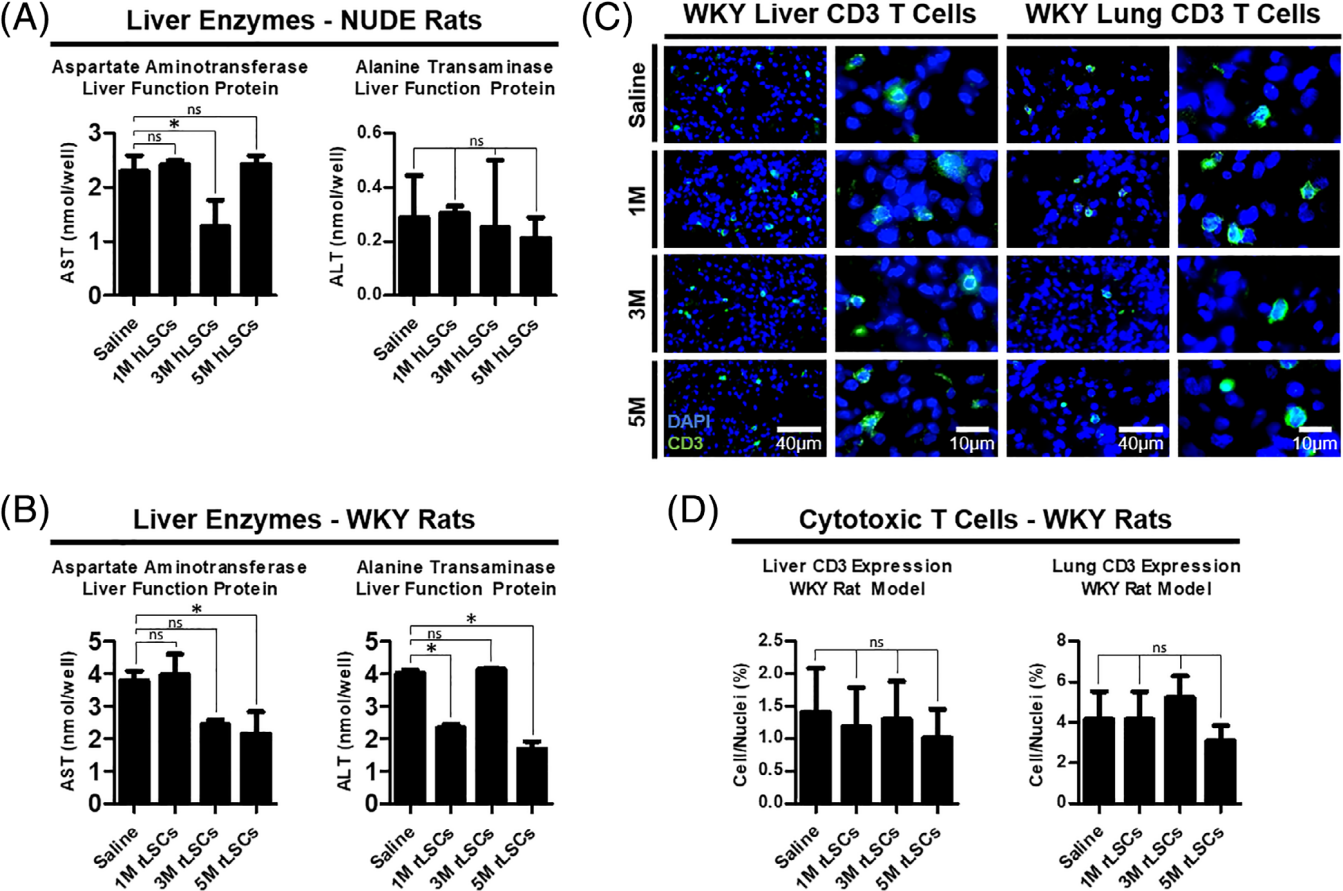
Safety study; liver toxicity and assessment of local lung rejection of LSC injections. A, Bar graphs summarizing the AST (left) and ALT (right) protein levels in WKY blood plasma, n = 3. B, Bar graphs summarizing the AST (left) and ALT (right) protein levels in nude blood plasma, n = 3. C, Representative fluorescent micrographs showing the dose‐dependent expression of CD3^+^ T cells in the WKY rat livers and lungs. D, Bar graphs summarizing the CD3 expression levels in each WKY treatment group, n = 3. Statistical analysis: one‐way analysis of variance with Bonferroni's multiple comparison test. Error bars represent SD. *P ≤ .05. M, million; ns, no significant difference; ALT, alanine transaminase; AST, aspartate aminotransferase; CD, cluster of differentiation; WKY, Wistar‐Kyoto

We tested the blood plasma of both rat breeds, per dosing group, for the proteins alanine transaminase (ALT) and aspartate aminotransferase (AST). Both enzymes are analyzed routinely in laboratory tests to determine liver health, especially ALT, which is predominantly produced in the liver. High levels of ALT can also be indicative of certain obesity related diseases, metabolic syndromes, and heart conditions.^32^ AST is predominantly produced in the heart, but also in the liver, and is sometimes the predominant marker used to detect cirrhosis.^33^ We found no significant increase in AST or ALT for either the 1, 3, or 5 million cells groups in comparison to their respective saline groups for either breed (Figure 6A,B). The AST and ALT values are summarized in Figures S17 and S18.

As an added precaution, we looked at the immune reaction behavior of the liver and lung tissues of all the WKY rat groups, by assessing in situ CD3^+^ T‐cell counts (Figure 6C,D). There was no significant difference between the T‐cell populations of the cell dose groups compared to the saline controls.

## 3 DISCUSSION

The alveoli and the bronchioalveolar junctions are composed of ciliated and nonciliated bronchiolar epithelial, club, alveolar type I epithelial, alveolar type II epithelial, smooth muscle, fibroblast, pericyte, and macrophage cells. Among these cell types, a number of progenitor cells have been identified.^34^, ^35^, ^36^, ^37^, ^38^ Type II alveolar epithelial cells (AECs), identified by SFTPC secretion, can divide and regenerate themselves or differentiate into type I AECs. Club cells positive for CCSP have been shown to give rise to type I and II AECs after bleomycin injury.^34^, ^39^ Putative CCSP and SFTPC bronchioalveolar stem cells in the bronchioalveolar junctions differentiate into type I and II AECs in vitro.^40^ They have also been shown to dedifferentiate back to CCSP secretory club cells. Other possible progenitors include Putative Integrin Beta 4^+^ and SFTPC^−^ AECs [13], and Trp63^+^ and Krt14^+^ basal cells.^41^ The cell types identified in the LSC cocktails contain SFTPC and CCSP positive progenitor cells, as well as type I AECs (AQP5), and mesenchymal/stromal supporting cells (CD105 and CD90).

An unexpected phenomenon that characterizes these cells is their coexpression of multiple phenotypic markers, which is made evident in Figures 3E,F. Although it is not definitively clear why this coexpression occurs, Liebler et al and Matsuzaki et al present evidence for two plausible reasons.^42^, ^43^ Liebler et al show that there are transitional cells in the lungs that coexpress markers present in both AT2 (SFTPC) and AT1 (AQP5) cells. These transitional cells may be part of the reason there is coexpression in the flow cytometry analysis. In addition, Matsuzaki et al provide a possible explanation for why the stromal‐mesenchymal markers (CD105 and CD90) are coexpressed with AEC markers. As explained therein, when cells are cultured on plastic surfaces, some cells may experience an epithelial to mesenchymal transition and begin to express markers that reflect the mesenchymal phenotype.

The cells undergo a sequential 2D‐3D‐2D culture system that is outlined in Figure S6. During the 3D culture stage, the cells self‐agglomerate into spheroids, in suspension, providing a more biomimetic growth environment. During this time, their CCSP and SFTPC protein expression levels are enhanced (Figure S7), resulting in a cell cocktail with a higher expression of progenitor markers. Thus, this brief 3D environment (4‐10 days of culture) is an important step in the manufacturing of LSCs. The synergy of all the cells in the cocktail, working together, releasing paracrine signals and communicating in a more biomimetic way, is likely responsible for the therapeutic effects.^44^, ^45^

The growth potentials and antigenic profiles of fibrosis‐derived hLSCs and rLSCs were assessed and compared to previous studies using nonfibrosis‐derived cell batches. IPF hLSCs have higher levels of CD105 and lower levels of CD90, AQP5, and SFTPC, but similar levels of CCSP, compared to their nonfibrosis‐derived counterparts; a thorough characterization of LSC composition is provided in Dinh et al.^14^ Some variability in the expression of each marker can be expected even between IPF human cell donors, especially given the heterogeneous nature of IPF and the natural variability involved in performing the biopsies. Quantitative measurements herein, obtained via flow cytometry, are focused on the IPF hLSCs, as this is the cell type that will be used in the clinical trial. A qualitative, histology‐based overview of phenotype was done on the rLSCs, although previous publications have characterized rLSCs using flow cytometry as well.^23^ The results demonstrate the conservation of the characteristic LSC markers across the two species. There will also be varying growth rates among the cell lines. Data for the total time it takes the cells to mature are provided in Figure 3C,D.

The persistence of fibrosis 30 days after bleomycin injections allowed for the adoption of a 20‐day timeline (Figure 4A) for the subsequent dosing study. Data were measured 10 days after cellular injections to simulate a 1 year therapeutic period in humans, given the conversion of rat to human years for adolescent rats.^46^ Rat cell doses were converted to human equivalents using the dose factor method,^47^ which takes into consideration the surface area and metabolic rates of the species being used (rat and human). The formula (Figure S1), adapted for our cell dosage study, yielded the following rat to human conversions: a 1 × 10^6^ and a 3 × 10^6^ cell injection in rats scales up to 68 × 10^6^ and 203 × 10^6^ cells in humans (Figure S12). For simplicity, the clinical trial low and high doses will be 100 × 10^6^ and 200 × 10^6^ hLSCs, respectively. To demonstrate time, cost, and manufacturing feasibility, 202 million cells were grown by the end of hLSC passage 3. In addition, the doses proposed herein for human clinical trials align with or are below those already being used in similar clinical studies.^48^, ^49^, ^50^, ^51^, ^52^, ^53^, ^54^

Two improvements that would strengthen these studies, and are future research aims, are an increase in rodent n‐values and obtaining functional data to corroborate the therapeutic efficacy of the cell injections. The first can be achieved by lowering the bleomycin potency from 4 to 2 or 3 U/kg. The higher the potency, the higher the likelihood that the rodent will die before all data points can be obtained. Although this might create models that are more susceptible to fibrotic self‐resolution, it would be worth the added statistical value to find a middle ground between model longevity and rodent mortality. It is worth noting that, despite the imperfections of the bleomycin‐induced IPF model, including reported self‐resolution of fibrosis and the rapid onset of the fibrotic development, the American Thoracic Society considers it the best characterized model available for preclinical testing, and it is certainly the most widely used in the literature.^55^ The latter can be achieved by making changes to the study design in future iterations. The evaluation of lung function became quite difficult for the nude and WKY. An initial attempt at gathering repeated lung function measurements in pilot studies proved that repeated dosing with Ketamine/Xylazine, in addition to Isoflurane (partial diaphragm paralysis), was too taxing on the health of the rats. Moreover, the acquisition of functional data required their intubation at frequent intervals, causing tracheal irritation and swelling. Follow‐up studies will measure lung function using a hardier rat breed, so that the anesthetic/intubation issues can be overcome.

This study provides an array of safety assessments that add to previously published research showing the general innocuousness of LSC therapy.^14^, ^23^ In this study, no tumorigenic growths were detected and dosing thresholds did not cause adverse effects. The liver, which plays a major role in all metabolic processes, was not harmed (based on AST and ALT levels), and the native lung parenchyma did not react immunogenically. Considering the results within the parameters of the animal models presented herein, in addition to the studies that predate this one and support it, this research sets the groundwork for a first in human LSC clinical study.

## 4 CONCLUSION

We have demonstrated the safety and efficacy of LSCs derived from fibrotic lungs in attenuating the severity of PF in two rat models of the disease. The fibrotic progression as a result of bleomycin injury in these rat models has been tracked to insure LSC injections are administered during the fibrotic stage of the disease. This is the first time rodent PF has been treated using transbronchial samples of hLSCs injected intravenously. This study sets the stage for the transition of this cellular therapy paradigm from rodent models to clinical trials in a number of ways: the transbronchial acquisition of the LSCs is a minimally invasive strategy suitable for the clinic and preferable to thoracoscopic alternatives; the doses used in this study are scalable, manufacturable, and comparable to currently existing clinical trial efforts targeting other lung diseases; and the intravenous route of administration used is applicable to clinical trials, as it presents an easy, quick, and patient‐friendly way to administer the treatment. The injections administered to our rodents led to a dose‐dependent improvement in fibrotic thickening and less ECM deposition, with no tumorigenic behavior in situ and no toxic reactions in the body. The safety and efficacy of LCSs will be tested in patients as part of a phase I, randomized, standard‐care‐controlled study set to begin patient recruitment in 2020.

### 4.1 Statistics

All results are presented as means ± SD unless otherwise specified. Comparisons between any two groups were performed using two‐tailed unpaired Student's *t* tests with a 95% confidence interval. One‐way analysis of variance was used to compare means among more than two groups, followed by post hoc Bonferroni correction. Statistical significance was achieved at *P* < .05.

### 4.2 Study approval

All animal work was compliant with the Institutional Animal Care and Use Committee at North Carolina State University.

## CONFLICT OF INTEREST

P.‐U.D. declared consultancy for with BreStem Therapeutics Inc. K.C. declared stock ownership interest in BreStem Therapeutics. The other authors declared no potential conflicts of interest.

## AUTHOR CONTRIBUTIONS

J.C.: conception and design of the studies, acquisition, analysis, and interpretation of data, and drafting and revisions; P.‐U.D., T.H.: acquisition, analysis, and interpretation of data, and drafting and revisions; K.B.A., L.J.L., K.C.: conception and design of the studies, and drafting and revisions.

## Supporting information


**Appendix** **S1.** Supporting Information.

**Appendix** **S2.** Supporting Figures.

## Data Availability

The data that support the findings of this study are available from the corresponding author upon reasonable request.
