# Supplementary material for: A pre‐investigational new drug study of lung spheroid cell therapy for treating pulmonary fibrosis
Source: Stem Cells Transl Med. 2020 Apr 18;9(7):786–98. doi: 10.1002/sctm.19-0167 (PMC7308638; doi:10.1002/sctm.19-0167)
Supplement: Supplementary file 1 — Appendix S1. Supporting Information. [file SCT3-9-786-s001.docx]

**Methods**

**Animal Procedures**

To induce pulmonary fibrosis, the rats were first weighed to establish a baseline. They were then intratracheally injected with 300 µl of bleomycin at a potency of 4U/kg of body weight. Body weights were averaged for simplicity. To track fibrotic progression, three rats from each breed were sacrificed at time points 7, 10, 14, and 30 days. At each excision point, 2 mL of blood were drawn from each rat for future plasma analysis. In addition to the blood draw, each rat underwent a pulmonectomy. Pulmonary tissue was preserved for histology and protein assays. Three rats from each breed were used as sham controls. These were manipulated in the same manner as the test subjects but received no injection. Three rats from each breed were used as saline controls. These were manipulated in the same manner as the test subjects but received tracheal injections of phosphate buffered saline instead of bleomycin. All rats were weighed periodically up until their end points. To find the minimum effective cell dose, rats were intravenously injected with either 1, 3, or 5 million LSCs 10 days after bleomycin injury.

**Bleomycin Administration**

The pharmacological dose per rat is weight dependent and measured in potency per kilogram of body weight (U/kg). The target potency value is 4U/kg and was obtained per rat using the following formulas:

| 1. | Desired Potency  (U/kg body weight) | x | Animal Weight  (kg) | = | Potency per Rat  (U/Rat) |
| --- | --- | --- | --- | --- | --- |
| 2. | Potency per Rat  (U/Rat) | x | Bleomycin Sulfate Potency  (mg/U) | = | Bleomycin Injection  (mg/Rat) |

The final amount (mg) of Bleomycin was dissolved in 300 µl of phosphate-buffered saline (PBS) solution. This full volume was injected into the rats intratracheally. The rats were first intubated with a 14-gauge catheter tube. The solution was injected into the tracheal passage and then the lungs. Intubation ensures consistency of bleomycin dosing and prevents spill-over into the esophagus.

**FlexiVent Procedures**

Rats were anesthetized with 1µl of a 2:1 Ketamine to Xylazine (KX) solution per gram of body weight. They were then intubated with 14-gauge catheters before being placed onto the FlexiVent plethysmography platform and connected to the ventilator module (Fig. 1E). Each rat was ventilated for a minimum of 1 minute with Isoflurane gas to ensure temporary lung paralysis, after which they were ventilated with room air. A standard script was run in triplicates for 3 minutes. The operations used were Deep Inflation, Snapshot-90, Quick Prime-3, Pressure Volume Loops (PVs-P), and Negative Pressure Forced Expirations (NPFE). The data output from these operations included inspiratory capacity (IC), respiratory system compliance (Crs), static compliance (Cst), forced vital capacity (FVC), and FEV0.2/FVC ratio. The rats were then subjected to a lung expansion and continued ventilation for a minute before being removed from the respirator. They were supervised until independent, regular breathing-patterns returned.

**Rat Tail-Vein Injections**

Rats were anesthetized with 300 µl of a 2:1 Ketamine to Xylazine solution and secured onto a surgical platform. A 31-gauge insulin syringe (Becton Dickinson, Franklin Lakes, NJ, http://www.bd.com/en-us) was used to inject 300 µl of either PBS + heparin (9:1) solution or cell suspension (also carried in PBS + heparin) into one of the two lateral tail veins. 1, 3, or 5 million cell were injected. The formula used to obtain the Human Equivalent Dose (HED) is explained in Supplemental Figure 1.

**Cell Culture**

The human IPF LSC line acquired for this study (Supplemental Figure 2) was isolated from human lung biopsies obtained from an IPF patient at UNC Medical Center. Three transbronchial biopsies were used for cell outgrowth and expansion. The rat PF LSC line was outgrown and expanded from fibrotic (bleomycin-induced) lung tissue excised from Wistar Kyoto rats. 0.5-1 mm diameter samples of distal lung tissue were separated and washed with phosphate-buffered saline (Life Technologies, Carlsbad, CA, http://www.lifetechnologies.com) to remove excess blood. The following protocol applies to both human and rat lung samples: The tissues were enzymatically digested at 37°C in 5 mg/ml collagenase type IV solution (Sigma-Aldrich, St. Louis, MO, http://www.sigmaaldrich.com). Collagenase digestion was inactivated after 5 minutes using an equal volume of Iscove’s modified Dulbecco’s medium (IMDM; Life Technologies) containing 20% fetal bovine serum (FBS; Corning Life Sciences, Acton, MA, http://www.corning.com/). Subsequently, the tissue samples were plated onto 150 mm fibronectin-coated petri dishes in 2 mL of 20% FBS IMDM overnight to allow for tissue-plate adhesion. The next day, 20 mL of media was added into the dish. The tissues were kept in media for one to two weeks (or until cells began to outgrow from the tissue explants) during which media was changed once every other day. At a confluence of 70%-80%, outgrowth cells were harvested from the petri dishes via 5-10 minutes of incubation with TryPLE Select (Life Technologies). The cells were passaged into ultra-low attachment flasks (Corning Life Sciences) at a density of 100,000 cells per cm^2^ and cultured with 10% FBS IMDM. Within 24-36 hours, spontaneous spheroid formation was observed and allowed to mature for up to a week. Lung spheroids were then collected and replated onto fibronectin-coated flasks (Corning Life Sciences) to generate lung spheroid cells (LSCs). These were cultured in 20% FBS IMDM containing 50 mg/ml gentamicin (Life Technologies), 2 mmol/L L-glutamine (Life Technologies), and 0.1 mmol/L 2-mercaptoethanol (Life Technologies).

**Residual Fetal Bovine Serum and Collagenase Testing**

The residual FBS levels in the cell culture media were measured by testing the cell culture supernatant levels of bovine transferrin, using an enzyme linked immunosorbent assay (ELISA) (E10-122; Bethyl Laboratories, INC, Montgomery, TX), according to the manufacturers protocol. Absorbance measurements were made at a wavelength of 450 nm. The residual collagenase levels in the cell culture media were measured by testing the activity of the enzyme with a Collagenase Activity Assay Kit (ab196999; Abcam), according to the manufacturers protocol. Absorbance measurements were made at a wavelength of 354 nm.

**Immunocytochemistry on Lung Spheroid Cells**

LSCs were plated onto fibronectin-coated 4-chamber Millicell® culture slides (EMD Millipore, Billerica, Massachusetts, http://www.emdmillipore.com). They were fixed with 4% paraformaldehyde (PFA) and blocked/permeabilized with Dako Protein Block Solution (Dako, Carpinteria, CA, http://www.dako.com) containing 0.1% saponin. Subsequently, the cells were treated with anti CD105, CD90, SFTPC, CCSP, and Aquaporin 5 antibodies overnight at 4°C, followed by incubation with Alexa Fluor 488, FITC, or Texas Red conjugated secondary antibodies (Abcam, Cambridge, MA, http://www.abcam.com) for microscopy. Images were taken with an epifluorescent microscope (Olympus IX81; Olympus, Center Valley, PA, http://www.olympusamerica.com). Antibody product details are provided in Supplemental Figure 3.

**Organ Histology**

Immunofluorescence (IF) was performed on 4% paraformaldehyde-fixed lung cryosections (5μm). Samples were permeabilized and blocked with Dako Protein Block Solution containing 0.1% saponin at room temperature for one 1 hour and then incubated with primary antibodies overnight at 4°C. The samples were then incubated with secondary antibodies for 1.5 hours at room temperature. 4',6-diamidino-2-phenylindole (DAPI) was applied for 10 min before mounting. All wash steps were performed with non-sterile PBS. The following antibody was used for tissue IF: CD3 (T Cell). Product information and secondary antibody paring is available in Supplemental Figure 3. In addition, paraffin preserved cryosections were stained with Hematoxylin and Eosin for Ashcroft scoring and Gomori trichrome for collagen detection at the Histology Core of the NCSU Veterinary Medicine College. Masson’s trichrome staining was conducted in lab by study researchers. Ashcroft, porosity, and trichrome scoring was conducted by multiple researchers blinded to group assignment.

**Flow Cytometry**

Human LSCs were incubated with anti CD105, CD90, SFTPC, CCSP, AQP5 antibodies, to confirm phenotypic consistency to previously published human LSC groups.^22,23^ Product information and secondary antibody paring is available in Supplemental Figure 3. Cell membranes were fixed and permiabilization (Cytofix/CytopermTM; BD Biosciences, San Diego, CA, http://www.bdbiosciences.com) prior to SFTPC and CCSP antibody incubation. All antibodies were incubated with 5 x 10^5^ LSCs in 300µl flow buffer for 60 minutes at 4°C in a 96 well plate. All markers were run in triplicates. Non-labeled cells were used as negative controls and isotype-identical antibodies served as non-specific binding controls. All flow cytometry was performed on a CytoFlexTM Flow Cytometer (Beckman Coulter, Brea, CA, https://www.beckmancoulter.com) and all data were analyzed with FCS Express software (De Novo Software, Glendale, CA, https://www.denovosoftware.com). The human LSCs were flowed without sorting by type. Each run was conducted using the total LSC cocktail. One marker was tested during each run.

**Protein Extraction and Quantification**

Total protein concentration was determined by BCA assay (Pierce, 23227) performed in triplicates using 10 μl of protein isolate for each run. Absorbance was measured at 562 nm on a plate reader and averaged.

**Investigation of Protein Expression through Western Blot Assay**

Lung protein isolates were reduced by β-mercaptoethanol and denatured at 100°C for 5 minutes. Proteins, run in triplicates, and a molecular weight standard (Bio-Rad, Precision Plus Protein Unstained Standards MW Ladder 161-0363) were separated by gel electrophoresis carried out on a 4-15% Tris-Glycine stain-free gel (Bio-Rad). The gels were run at a stack voltage of 100V for 5 minutes followed by a constant 200V for 30-40 minutes. The gels were activated and visualized by UV light in a ChemiDoc XRS+ Imager (Bio-Rad, Hercules, California). Wet transfers were conducted using the Bio-Rad Mini-PROTEAN Tetra Cell system. After the transfer, the membranes were washed three times in PBS-T, then blocked using 5% milk in PBS-T for 1 hr. The primary INF-gamma antibody was incubated overnight at 4°C. A secondary goat anti-rabbit HRP-conjugated antibody was incubated for 1 hour at room temperature. Antibody information is available in Supplemental Figure 3.

**Liver Function Assays**

Two colorimetric assays were used to analyze liver health; the Aspartate Aminotransferase Activity Assay Kit (Abcam, ab105135), and the Alanine Transaminase Activity Assay Kit (Abcam, ab105134). Each assay was performed according to protocol specifications. Optical Density was measured using a Synergy Neo 2 multi-mode reader (BioTek, Winooski, VT, https://www.biotek.com).
