# Supplementary material for: A pre‐investigational new drug study of lung spheroid cell therapy for treating pulmonary fibrosis
Source: Stem Cells Transl Med. 2020 Apr 18;9(7):786–98. doi: 10.1002/sctm.19-0167 (PMC7308638; doi:10.1002/sctm.19-0167)
Supplement: Supplementary file 2 — Appendix S2. Supporting Figures. [file SCT3-9-786-s002.docx]

**Supplemental Figures**

$${HED \left( \frac{mg}{kg} \right)=Animal NOAEL \left( \frac{mg}{kg} \right)x(\frac{Animal Weight\left[ kg \right]}{Human Weight \left[ kg \right]})}^{(1-0.67)}$$

$${HED \left( \frac{cells}{kg} \right)=Animal NOAEL \left( \frac{cells}{kg} \right)x(\frac{Animal Weight\left[ kg \right]}{Human Weight \left[ kg \right]})}^{(1-0.67)}$$

**Supplemental Figure 1**: The formula used to obtain the Human Equivalent Dose (HED) uses the animal dose/level at which there are no observed adverse effects (NOAEL). This formula, shown above, was adapted for our cell dosage study. The units used in our formula are cells/kg instead of mg/kg. The average weights of our Nude and WKY rats before bleomycin injections were 147g and 159g, respectively. We used an average of 150g per rat in the formula for simplicity. The average weight of an adult in North America is 81kg (Centers for Disease Control and Prevention (CDC)).

| **Supplemental Figure 2: Human LSC Donor Information** | | | | | |
| --- | --- | --- | --- | --- | --- |
|  | **Sex** | **Age** | **Race** | **Smoker** | **Additional Information** |
| **TB Donor 1** | Male | 46 | Hispanic | No | Fibrosing NSIP; Received Lung Transplant |
| **TB Donor 2** | Male | 76 | White | No | Mixed Connective Tissue Associated NSIP |
| **TB Donor 3** | Male | 64 | White | Yes | IPF; Received Lung Transplant |

**Supplemental Figure 2**: Details of the human pulmonary cell lines used for Nude rat injections (Transbronchial (TB) Donor 3) and in-vitro assays (TB Donors 1-3). NSIP: Non-specific interstitial pneumonia, IPF: Idiopathic Pulmonary Fibrosis.

| **Supplemental Figure 3: ICC, IHC, & Flow Cytometry Antibody Details** | | | |
| --- | --- | --- | --- |
| **Primary Antibodies** | **Product Number** | **Secondary Antibodies** | **Product Number** |
| AQP5 | ab78486 | Alexa Fluor 488 | A11034 |
|  |  | Texas Red | ab6787 |
| AQP5 | sc9890 | FITC | ab7121 |
| SFTPC | bs10067R | FITC | Pre-Conjugated |
| CCSP | ab40873 | Alexa Fluor 488 | A11034 |
| CD90 | ab226 | FITC | Pre-Conjugated |
| CD90 | ab23894 | Alexa Fluor 488 | A10667 |
|  |  | Texas Red | ab6787 |
| CD3 | ab16669 | Alexa Fluor 488 | A11034 |

**Supplemental Figure 3**: Summary of antibodies used for cytochemistry (ICC), immunohistochemistry (IHC), and flow cytometry.

| **Supplemental Figure 4: Inflammation – WKY Rats** | | | | | |
| --- | --- | --- | --- | --- | --- |
|  | **Sham** | **Day 07** | **Day 10** | **Day 14** | **Day 30** |
| **Mean** | 588926 | 765585 | 1.426e+006 | 1.436e+006 | 1.052e+006 |
| **Std. Deviation** | 139619 | 174460 | 130595 | 75172 | 57731 |
|  | **Statistical Significance P < 0.05** | | | | |
| **D07 vs D10** | Yes | | | | |
| **D07 vs D14** | Yes | | | | |
| **D07 vs D30** | No | | | | |
| **D07 vs Sham** | No | | | | |
| **D10 vs D14** | No | | | | |
| **D10 vs D30** | Yes | | | | |
| **D10 vs Sham** | Yes | | | | |
| **D14 vs D30** | Yes | | | | |
| **D14 vs Sham** | Yes | | | | |
| **D30 vs Sham** | Yes | | | | |

**Supplemental Figure 4:** Summary of WKY inflammatory values and statistics.

| **Supplemental Figure 5: Flow Cytometry Cell Validation Results** | | | | | | | | |
| --- | --- | --- | --- | --- | --- | --- | --- | --- |
| **BX#** | **Phenotype** | **Cell yield at LSC P1** | **Lot release Viability** | **In-process Sterility** | **Lot release sterility** | **In-process Mycoplasma (PCR)** | **Lot release Mycoplasma**  **(PTC 1993)** | **Lot release Endotoxin** |
| HL052 | CD105^+^ = 88.3%,  CCSP^+^ = 64.12%,  AQP5^+^ = 67.0%  SFTPC^+^ = 35.0%  CD45^+^ = 6.1% | 12x10E6 | 98.5% | Negative | Negative | Negative | Negative | Negative |

**Supplemental Figure 5**: Summary of Human IPF LSC phenotypic markers.

**
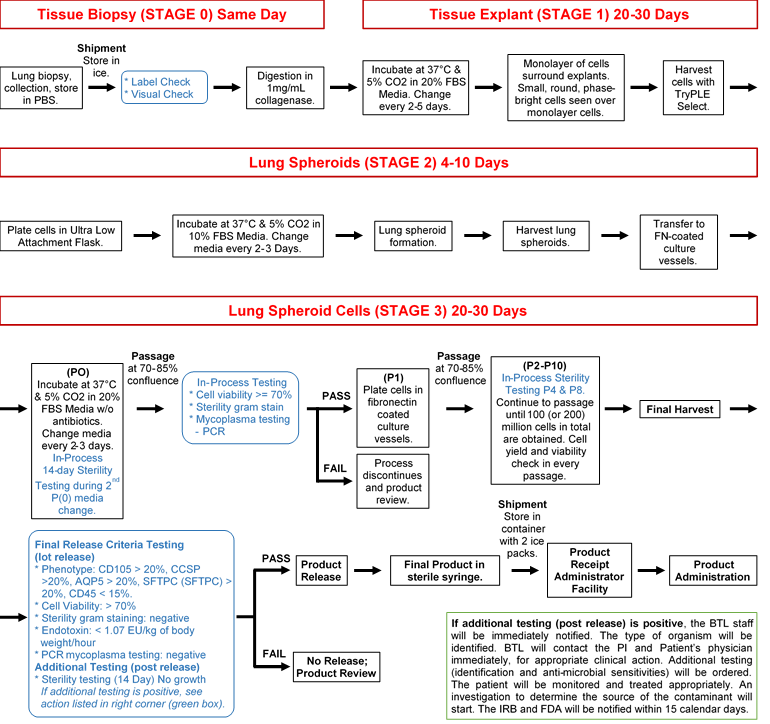
**

**Supplemental Figure 6**: Summary clinical cell-expansion protocol.

**
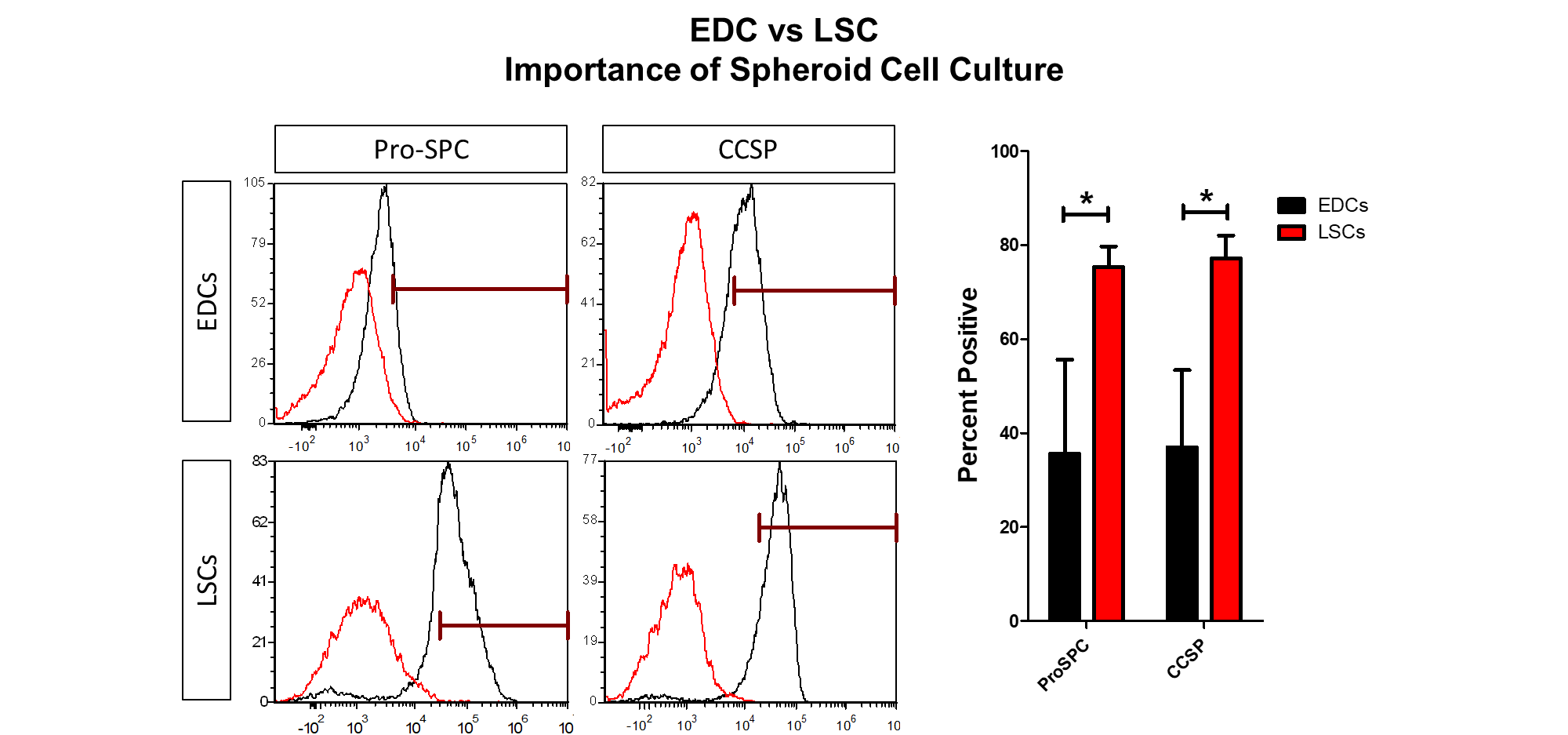
**

**Supplemental Figure 7**: Flow cytometry data for alveolar epithelial type II cells (SFTPC) and club cells (CCSP) expression in EDCs and LSCs.

| **Supplemental Figure 8: Human LSC Expansion** | | | | | |
| --- | --- | --- | --- | --- | --- |
| Passage 1  **(1 Flask)**  **Day 0** | Viability | Passage 2  **(5 Flasks)**  **Day 5** | Viability | Passage 3  **(20 Flasks)**  **Day 14** | Viability |
| **18.6 x 10^6^ Cells** | **92%** | **75.2 x 10^6^ Cells** | **93%** | **201.9 x 10^6^ Cells** | **81%** |

**Supplemental Figure 8**: Summary of Human LSC expansion, viability, timeline, and T-175 Flask requirements.

**
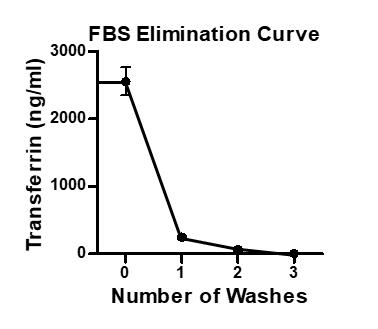
**

**Supplemental Figure 9**: Line graph showing the decrease in transferrin and, by extension, fetal bovine serum, in cell culture media, after three washes.

| **Supplemental Figure 10: FBS Removal** | | | |
| --- | --- | --- | --- |
| **Sample** | **First Wash**  **Calculated % FBS** | **Second Wash**  **Calculated % FBS** | **Third Wash**  **Calculated % FBS** |
| Sample#1 | 0.01004% | 0.00250% | <0.00016% |
| Sample#2 | 0.00724% | 0.00310% | <0.00067% |
| Sample#3 | 0.00838% | 0.00148% | <0.00001% |

**Supplemental Figure 10**: Summary of calculated residual FBS percentages in cell supernatant. FBS values were calculate from Transferrin quantities in the cell supernatant.

**
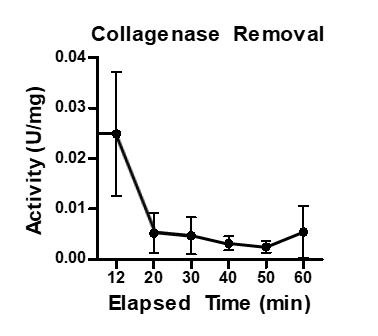
**

**Supplemental Figure 11**: Line graph showing the decrease in collagenase activity in cell culture media.

| **Supplemental Figure 12: Rat to Human Conversions** | |
| --- | --- |
| **Rat Dose** | **Human Dose** |
| 1 Million | 68 Million |
| 3 Million | 203 Million |
| 5 Million | 339 Million |

**Supplemental Figure 12**: Conversion chart between rat and human doses.

| **Adverse Events Nude Rats** | **Baseline**  **Pre-Bleo** | **During Bleo Procedure** | **Fibrotic Buildup Period** | **During Cell Infusion** | **Cell Infusion to Sacrifice** |
| --- | --- | --- | --- | --- | --- |
| **1x10^6^ IPF LSC Dose** |  |  |  |  |  |
| C236R1 | None | None | LB | LB | LB |
| C237R1 | None | None | LB | LB | LB |
| C240R2 | None | None | LB | LB | LB |
| **3x10^6^ IPF LSC Dose** |  |  |  |  |  |
| C238R1 | None | None | LB | LB | LB |
| C239R1 | None | None | LB | LB | LB |
| C241R2 | None | None | LB | LB | LB |
| **5x10^6^ IPF LSC Dose** |  |  |  |  |  |
| C240R3 | None | None | LB | LB | LB |
| C241R3 | None | None | LB | LB | LB |
| C242R2 | None | None | LB | LB | LB |
| C239R2 | None | None | LB | LB; DPI | N/A |
| C239R3 | None | None | LB | LB; DPI | N/A |
| C240R1 | None | None | LB | LB; DPI | N/A |
| **PBS Control** |  |  |  |  |  |
| C237R3 | None | None | LB | LB | LB |
| C238R3 | None | None | LB | LB | LB |
| C241R1 | None | None | LB | LB | LB |

* Death Post Injection (DPI); Very Weak (VW); Labored Breathing (LB)

* Labored breathing and some weakness is expected after bleomycin is introduced

**Supplemental Figure 13**: Summary of adverse events recorded for Nude rats in the dosing studies.

| **Adverse Events**  **WKY Rats** | **Baseline**  **Pre-Bleo** | **During Bleo Procedure** | **Fibrotic Buildup Period** | **During Cell Infusion** | **Cell Infusion to Sacrifice** |
| --- | --- | --- | --- | --- | --- |
| **1x10^6^ IPF LSC Dose** |  |  |  |  |  |
| C250R1 | None | None | LB | LB | LB |
| C250R2 | None | None | LB | LB | LB |
| C252R2 | None | None | LB | LB | LB; VW |
| C243R1 | None | Died | --- | --- | --- |
| C245R2 | None | Died | --- | --- | --- |
| **3x10^6^ IPF LSC Dose** |  |  |  |  |  |
| C243R2 | None | None | LB | LB | LB |
| C244R1 | None | None | LB | LB | LB |
| C244R2 | None | None | LB | LB | LB |
| C247R2 | None | None | LB | LB | LB |
| C245R1 | None | None | Died | --- | --- |
| **5x10^6^ IPF LSC Dose** |  |  |  |  |  |
| C246R1 | None | None | LB | LB | LB |
| C246R2 | None | None | LB | LB | LB |
| C249R2 | None | None | LB |  | LB; VW |
| C249R1 | None | Died | --- | --- | --- |
| C247R1 | None | Died | --- | --- | --- |
| **PBS Control** |  |  |  |  |  |
| C206R1 | None | None | LB | LB | LB |
| C206R2 | None | None | LB | LB | LB; VW |
| C248R1 | None | None | LB | LB | LB |
| C251R1 | None | Died | --- | --- | --- |
| C251R2 | None | Died | --- | --- | --- |

* Death Post Injection (DPI); Very Weak (VW); Labored Breathing (LB)

* Labored breathing and some weakness is expected after bleomycin is introduced

**Supplemental Figure 14**: Summary of adverse events recorded for WKY rats in the dosing studies.

| **Supplemental Figure 15: Pathology – Nude Rats** | | | | | | |  |
| --- | --- | --- | --- | --- | --- | --- | --- |
| **Animal ID** | **Tumor Formations** | | | | | | |
|  | **Heart** | **Liver** | **Lung** | **L. kidney** | **R. Kidney** | **Spleen** | |
| C236R1 | None | None | None | None | None | None | |
| C237R1 | None | None | None | None | None | None | |
| C240R2 | None | None | None | None | None | None | |
| C238R1 | None | None | None | None | None | None | |
| C239R1 | None | None | None | None | None | None | |
| C241R2 | None | None | None | None | None | None | |
| C240R3 | None | None | None | None | None | None | |
| C241R3 | None | None | None | None | None | None | |
| C242R2 | None | None | None | None | None | None | |
| C239R2 | None | None | None | None | None | None | |
| C239R3 | None | None | None | None | None | None | |
| C240R1 | None | None | None | None | None | None | |
| C237R3 | None | None | None | None | None | None | |
| C238R3 | None | None | None | None | None | None | |
| C241R1 | None | None | None | None | None | None | |
| C236R2 | None | None | None | None | None | None | |
| C236R3 | None | None | None | None | None | None | |
| C237R2 | None | None | None | None | None | None | |
| C238R2 | None | None | None | None | None | None | |
| C242R1 | None | None | None | None | None | None | |

**Supplemental Figure 15**: Summary of pathological tumorigenicity inspection for Nude rats in the dosing study.

| **Supplemental Figure 16: Pathology – WKY Rats** | | | | | | |  |
| --- | --- | --- | --- | --- | --- | --- | --- |
| **Animal ID** | **Tumor Formations** | | | | | | |
|  | **Heart** | **Liver** | **Lung** | **L. kidney** | **R. Kidney** | **Spleen** | |
| C206R1 | None | None | None | None | None | None | |
| C206R2 | None | None | None | None | None | None | |
| C243R2 | None | None | None | None | None | None | |
| C244R1 | None | None | None | None | None | None | |
| C244R2 | None | None | None | None | None | None | |
| C246R1 | None | None | None | None | None | None | |
| C246R2 | None | None | None | None | None | None | |
| C247R2 | None | None | None | None | None | None | |
| C248R1 | None | None | None | None | None | None | |
| C249R2 | None | None | None | None | None | None | |
| C250R1 | None | None | None | None | None | None | |
| C250R2 | None | None | None | None | None | None | |
| C252R2 | None | None | None | None | None | None | |

**Supplemental Figure 16**: Summary of pathological tumorigenicity inspection for WKY rats in the dosing study.

| **Supplemental Figure 17: Liver Function Proteins – Nude Rats** | | | | |
| --- | --- | --- | --- | --- |
| **AST** | **Saline Ctrl.** | **1x10^6^ hLSCs** | **3x10^6^ hLSCs** | **5x10^6^ hLSCs** |
| Mean | 2.29 | 2.41 | 1.27 | 2.42 |
| Std. Deviation | 0.30 | 0.07 | 0.49 | 0.16 |
|  | **Statistical Significance P < 0.05** | | | |
| Saline Ctrl. vs 1x10^6^ hLSCs | No | | | |
| Saline Ctrl. vs 3x10^6^ hLSCs | Yes | | | |
| Saline Ctrl. vs 5x10^6^ hLSCs | No | | | |
| **ALT** | **Saline Ctrl.** | **1x10^6^ hLSCs** | **3x10^6^ hLSCs** | **5x10^6^ hLSCs** |
| Mean | 0.29 | 0.30 | 0.25 | 0.21 |
| Std. Deviation | 0.16 | 0.03 | 0.25 | 0.08 |
|  | **Statistical Significance P < 0.05** | | | |
| Saline Ctrl. vs 1x10^6^ hLSCs | No | | | |
| Saline Ctrl. vs 3x10^6^ hLSCs | No | | | |
| Saline Ctrl. vs 5x10^6^ hLSCs | No | | | |

**Supplemental Figure 17**: Summary of AST and ALT liver enzyme values for Nude rats.

| **Supplemental Figure 18: Liver Function Proteins – WKY Rats** | | | | |
| --- | --- | --- | --- | --- |
| **AST** | **Saline Ctrl.** | **1x10^6^ rLSCs** | **3x10^6^ rLSCs** | **5x10^6^ rLSCs** |
| Mean | 3.78 | 3.97 | 2.44 | 2.16 |
| Std. Deviation | 0.31 | 0.63 | 0.14 | 0.68 |
|  | **Statistical Significance P < 0.05** | | | |
| Saline Ctrl. vs 1x10^6^ rLSCs | No | | | |
| Saline Ctrl. vs 3x10^6^ rLSCs | No | | | |
| Saline Ctrl. vs 5x10^6^ rLSCs | Yes (lower value than saline control) | | | |
| **ALT** | **Saline Ctrl.** | **1x10^6^ hLSCs** | **3x10^6^ hLSCs** | **5x10^6^ hLSCs** |
| Mean | 4.01 | 2.33 | 4.14 | 1.69 |
| Std. Deviation | 0.11 | 0.13 | 0.02 | 0.23 |
|  | **Statistical Significance P < 0.05** | | | |
| Saline Ctrl. vs 1x10^6^ rLSCs | Yes (lower value than saline control) | | | |
| Saline Ctrl. vs 3x10^6^ rLSCs | No | | | |
| Saline Ctrl. vs 5x10^6^ rLSCs | Yes (lower value than saline control) | | | |

**Supplemental Figure 18**: Summary of AST and ALT liver enzyme values for WKY rats.
